# Measuring cognitive and affective empathy across positive and negative emotions: psychometric properties and measurement invariance of the Perth Empathy Scale

**DOI:** 10.3389/fpsyt.2025.1533611

**Published:** 2025-03-11

**Authors:** Arezou Lashkari, Jack D. Brett, Ghasem Abdolpour, Mahdi Mazidi

**Affiliations:** ^1^ Department of Psychology, Faculty of Education and Psychology, University of Isfahan, Isfahan, Iran; ^2^ School of Psychological Science, The University of Western Australia, Crawley, WA, Australia; ^3^ The Kids Research Institute Australia, The University of Western Australia, Perth, WA, Australia; ^4^ Department of Psychology, Faculty of Humanities and Social Science, University of Mazandaran, Babolsar, Iran; ^5^ Centre for the Advancement of Research on Emotion, The University of Western Australia, Crawley, WA, Australia

**Keywords:** empathy, factor structure, measurement invariance, affective empathy, cognitive empathy

## Abstract

**Introduction:**

Empathy, a complex and multidimensional construct essential for social functioning and mental health, has been extensively studied in both research and clinical settings. The Perth Empathy Scale (PES), a recently developed self-report measure, assesses cognitive and affective empathy across both positive and negative emotions and is based on the self-other model of empathy. This study aimed to evaluate the psychometric properties of the Persian version of the PES in large Iranian sample.

**Methods:**

A total of 868 Iranian adults participated in this study. Factorial validity was examined through Confirmatory Factor Analysis (CFA).Internal consistency and concurrent validity were assessed by examining correlations with established empathy measures, including the Interpersonal Reactivity Index (IRI) and the Questionnaire of Cognitive and Affective Empathy (QCAE), as well as the Perth Alexithymia Questionnaire (PAQ) and the Social Phobia Inventory (SPIN). Measurement invariance was also tested between Iranian and Australian samples.

**Results:**

The findings supported a three-factor model comprising cognitive empathy, negative affective empathy, and positive affective empathy. The Persian PES demonstrated structural validity, internal consistency, and concurrent validity, suggesting it is a reliable measure for empathy assessment across cultural contexts.

**Discussion:**

This study provides empirical support for the validity of the Persian PES and contributes to the expanding body of research on empathy assessment. The results suggest that the PES can be effectively used in Persian- speaking populations.

Empathy is a psychological construct defined as an emotional reaction related to the emotions and situations of others, by matching their emotional states (1). Empathy has been considered a complex and multidimensional construct (2, 3). A large body of research claims that empathy is required for prosocial behavior (4) and is related to altruism and forgiveness (5, 6). Individuals with high levels of empathy are considered more cooperative and demonstrate more moral reasoning compared to those with lower levels of empathy (7). In addition to its role in promoting prosocial behaviors, the important role of empathy in clinical domains has been demonstrated too, with many studies showing that empathy is implicated in various disorders, such as social anxiety disorder (8), autism spectrum disorder (9), narcissistic personality disorder (10), and eating disorders (11). Given the significant role of empathy in social functioning and mental health, the accurate assessment of empathy is essential to inform both research and clinical practice. To date, many different measures have been developed to assess empathy (12). Recently Brett, Becerra (13) introduced five criteria that a comprehensive measure for the assessment of empathy should meet. Based on the contemporary theories of empathy, they proposed that a comprehensive empathy measure needs to meet the following criteria: (a) considers the self-other distinction, (b) assesses both cognitive and affective empathy, (c) assesses empathy for both negative and positive emotions, (d) considers emotion congruency between the target of empathy and the responder; and finally, (e) being psychometrically sound, meaning it should have acceptable internal consistency and good factor structure validity. In what follows, we briefly discuss what each of these criteria means and its importance for the assessment of empathy.

The first criterion for an optimal assessment of empathy is considering both self and others. This criterion is based on the well-established self-other model (14), which distinguishes the self and the other for showing empathy. The self-other model states that we deduce the affective states of others in two ways: the situation understanding system and the affective cue classification system. The first involves understanding the situation to provide an estimate of another person’s affective state based on the context in which they are. For example, seeing a black tie may indicate a funeral. The affective cue classification system includes interpreting facial expressions and tone of voice. Theorists of this model suggest that these two systems are necessary to create empathy. These two systems connect to the theory of mind and emotional representation systems. The affective cue classification system activates the mirror neuron system, which facilitates emotional contagion, such as automatic mimicry of emotional expressions (14). However, for an individual to experience affective empathy, it is essential they understand that the source of this sentiment is not from the individual himself, and hence the activation of the theory of mind and affective representation system is needed.

The next criterion that an optimal measure of empathy should meet is the assessment of both cognitive and affective aspects of empathy. Most researchers divide empathy into two dimensions: cognitive empathy and affective empathy. Cognitive empathy has been defined as understanding others’ experiences, while affective empathy is considered the ability to feel others’ emotional experiences (15). Recent research about taxonomy of empathy has explored the diversity in the definition of empathy through the assessment of 146 definitions of empathy (3). This research has reported a meta-definition of empathy as “the ability to experience affective and cognitive states of another person, while maintaining a distinct self, in order to understand the other”. This definition highlights the well-documented distinction between affective and cognitive empathy in the literature.

Another criterion that an optimal empathy assessment tool should meet is to distinguish between positive and negative emotions. Researchers have found that empathizing with positive and negative emotions has different neurological processes (16, 17). This distinction is clinically important too. For example, individuals with social anxiety disorder may feel anxious in the presence of others, which leads them to avoid social situations. These individuals can share others’ negative emotions although they are less able to share others’ positive emotions, compared to individuals without social anxiety disorders (18). Therefore, the tools used to assess empathy must distinguish between positive and negative emotions.

The fourth criterion that an optimal empathy assessment measure should meet is emotion congruency. Emotion congruency is essential for empathizing with others. Emotional congruency distinguishes empathy from other constructs such as sympathy and compassion. When the emotion experienced is the same as that of the other person, the individual experiences empathy. However, sympathy is related to the feelings of concern and sorrow about distressful situation in another person’s life. In other words, sympathy occurs when the individual feels for another (19, 20). Finally, an empathy assessment tool should demonstrate strong psychometric properties, including a theoretically supported factor structure, internal consistency, and expected associations with relevant constructs.

Brett, Becerra (13) identified 16 self-report measures specifically designed to assess empathy among adults in general. They examined these measures according to aforementioned five criteria and concluded that none of the existing measures fully meet all the criteria. A recent meta-analysis by Lima and Osório (12) indicated that the Empathy Quotient (EQ) (21), Interpersonal Reactivity Index (IRI) (22) and the Questionnaire of Cognitive and Affective Empathy (QCAE) (15) are the most commonly used measures to assess empathy. However, many of these commonly used tools such as the IRI and EQ do not separate the cognitive and affective dimensions of empathy. Additionally, most of these measures, including the QCAE, fail to distinguish between positive and negative valence of emotion. The two measures that differentiate the valence of emotions are the Cognitive Affective Somatic Empathy Scale (23, 24) and the Empathy Assessment Index (25), but they are not based on the self-other model of empathy.

To overcome these limitations, Brett, Becerra (13) developed the Perth Empathy Scale (PES). PES is a self-report measure that is based on the self-other theory of empathy and differentiates between the valence of emotions. The valence-specific assessment of PES results in four subscales. The first subscale is Negative Cognitive Empathy (NCE, five items, e.g., “Just by seeing or hearing someone, I know if they are feeling sad”). The second subscale is Positive Cognitive Empathy (PCE, five items, e.g., “Just by seeing or hearing someone, I know if they are feeling happy”). The two other subscales are Negative Affective Empathy (NAE, five items, e.g., “When I see or hear someone who is sad, it makes me feel sad too”), and Positive Affective Empathy (PAE, five items, e.g., “When I see or hear someone who is happy, it makes me feel happy too”).

Research has suggested that emotional functioning varies across different cultures (26–28). For instance, one study found that Asian participants exhibited lower affective empathy but higher cognitive empathy compared to British participants (27). However, cultural differences may bias measurement causing these differences instead of true empathic differences. Therefore, evaluating the psychometric properties of an instrument across different cultural contexts is essential. In fact, the benefit of measurement invariance is that it enables cross-cultural comparisons. If an instrument demonstrates different factor structures across two cultures, it cannot be reliably used to address questions about cultural differences. As a result, we have conducted measurement invariance analysis to compares empathy between the two cultures.

In addition to the original English version of the PES, the psychometric features of the PES have been examined in Chinese and Polish samples too, and the results supported the psychometric properties of the PES (29–32). However, to date no studies have examined the PES among Iranian samples. Therefore, the aim of the current study was to examine the psychometric properties of the Persian version of the PES among Iranian adults. We tested the PES for its factor structure, internal consistency, and concurrent validity. To test concurrent validity, relationships were examined between PES and the two subscales of the IRI (perspective-taking, empathic concern), the QCAE, the Perth Alexithymia Questionnaire (PAQ), and the Social Phobia Inventory (SPIN). In addition, we examined the cultural measurement invariance of the PES across Iranian and Australian adults.

## Materials and method

### Participants and procedure

Ethics approval for this project was granted by the Human Research Ethics Committees of both the University of Western Australia and the University of Isfahan in Iran. All participants provided informed consent for the use of their data.

#### Iranian adult sample

The Iranian adult general population sample consisted of 964 participants that were selected using a convenience sampling approach. They were recruited through advertisements posted on different social media platforms and completed the Persian version of the online survey via the Porsline platform (https://survey.porsline.ir/). Careless responding was also checked according to current guidelines (33, 34). Specifically, we checked instructed response items, which included any incorrect responses to three attention check items, and response time, noting instances where response time was less than 2 seconds for each item (13, 35). Ninety-six participants were excluded in quality screening. Furthermore, four participants were excluded as they reported being younger than 18 years old. The final sample consisted of 868 participants (86.5% female; mean age = 32.52, *SD* = 8.72, range = 18-72). Most of the respondents were married (60.5%, 298), 34.3% were single, and 4.8% respondents reported being divorced, while 0.3% reported that their spouse had died. The perceived socioeconomic status of the participants was measured using a ten-point Likert scale, where the first level represented the lowest perceived socioeconomic status, and the tenth level indicated the highest. The largest proportion of respondents rated themselves at level five (26.8%), followed by level four (21.3%), level three (16.1%), and level six (12.2%). The remaining levels collectively accounted for approximately 23% of the responses.

In terms of ethnicity, the majority of respondents (47.4%) were Fars, followed by Turk (23.4%), Kurd (6.1%), Gilak (5.3%), Lor (5.2%). The remaining respondents, comprising 12.8% of the sample, identified as Arab, Baluch, Turkmen, Mazani. The education level of the sample was as follows: 0.1% had elementary education, 3.3% had high school education, 37.4% had a diploma, 46.1% had a bachelor’s degree, 11.6% had a master’s degree and 1.4% had a Ph.D. degree. Iranian adult participants were given access to two monetary prize draws for their participation.

#### Australian adult sample

To permit assessing the measurement invariance of the PES across two cultures, we used Australian adult sample, too. We used an Australian adult sample collected for original study (13) to assess the measurement invariance of the PES across two cultures. The Australian sample consisted of 736 adults (68.2% female; mean age= 25.74, *SD*=10.71).

### Materials

#### Perth Empathy Scale (PES)

The PES is a 20-item self-report tool that measures empathy across both positive and negative emotions (13). Participants rate each item on a 5-point Likert scale (1 = Almost never; 5 = Almost always). In addition to the total scale score, the PES provides scores for four dimensions of empathy: NCE, PCE, PAE and NAE. Good convergent and discriminant validity, and internal consistency has previously been reported for the PES (13, 29, 30).

#### Translation and adaptation procedure of the PES

All PES items were translated into Persian using the standard back translation technique. First, a native Persian-speaker psychologist (the first author) translated the English version of the PES, which then was back-translated into English by an independent translator. The authors of this research held meetings with the developers of the PES to discuss the translation fluency and cultural adaptation, ensuring that the items were appropriately translated. After confirming the translation, we used the Persian version of PES in the study. Copies of both Persian and English versions of the PES, with scoring instructions are provided in the **Supplementary Materials**.

#### Questionnaire of Cognitive and Affective Empathy (QCAE)

The QCAE (15) is a 31-item self-report measure designed to assess both cognitive and affective empathy. The QCAE uses a 4-point Likert rating that ranges from strongly disagree (1) to strongly agree (4), with higher scores indicating a greater ability for empathy. The QCAE has been validated in multiple countries and demonstrated strong psychometric properties across different languages (15, 36, 37). Internal consistency of the QCAE in an Iranian sample for affective and cognitive empathy was 0.73 and 0.79, respectively (38). We used the QCAE scores for cognitive empathy and affective empathy.

#### Interpersonal Reactivity Index (IRI)

The IRI (22), is a 28-item questionnaire that is rated on a 5-point Likert scale ranging from does not describe me well (1) to does describe me very well (5). The IRI comprises four subscales: perspective–taking, empathic concern, fantasy, and personal distress. In the present study, we utilized the perspective-taking and empathic concern subscales, which are the most relevant to cognitive and affective empathy, respectively (39). The IRI is one of the most widely used empathy scales in the literature and has been validated in various cultures (40, 41). The Persian version of the IRI has also demonstrated good psychometric properties (42).

#### Perth Alexithymia Questionnaire (PAQ)

The PAQ is a 24-item self-report tool that measures alexithymia across both positive and negative emotions (43). Participants respond to each item on a 7-point Likert scale (1 = *strongly disagree*; 7 = *strongly agree*), with higher scores reflecting greater levels of alexithymia. In addition to the total scale score, the PAQ provides scores for five dimensions of alexithymia: N-DIF, P-DIF, N-DDF, P-DDF, and G-EOT. Previous research has demonstrated good convergent and discriminant validity, as well as strong internal consistency for the PAQ (43). The Persian version of the PAQ has indicated the same factor structure and robust psychometric properties (35, 44).

#### Social Phobia Inventory (SPIN)

The SPIN is a 17-item self-report measure used to assess social anxiety (45). Each item is rated on a scale from 0 (not at all) to 4 (extremely), with higher scores indicating greater social anxiety. The full scale ranges from 0 to 68. The SPIN has demonstrated strong psychometric properties, including test-retest reliability, internal consistency, convergent validity, divergent validity, and construct validity as reported in various studies (45, 46). The Persian version of the SPIN has demonstrated strong psychometric properties (47). We included the Social Phobia Inventory (SPIN) in the present study based on evidence suggesting that individuals with social anxiety may exhibit asymmetries in empathic responses (18), as well as the recommendation from the developers of the PES in the original study to incorporate a social anxiety measure given its theoretical relevance to empathy (13).

### Analytic strategy

**Figure 1:**
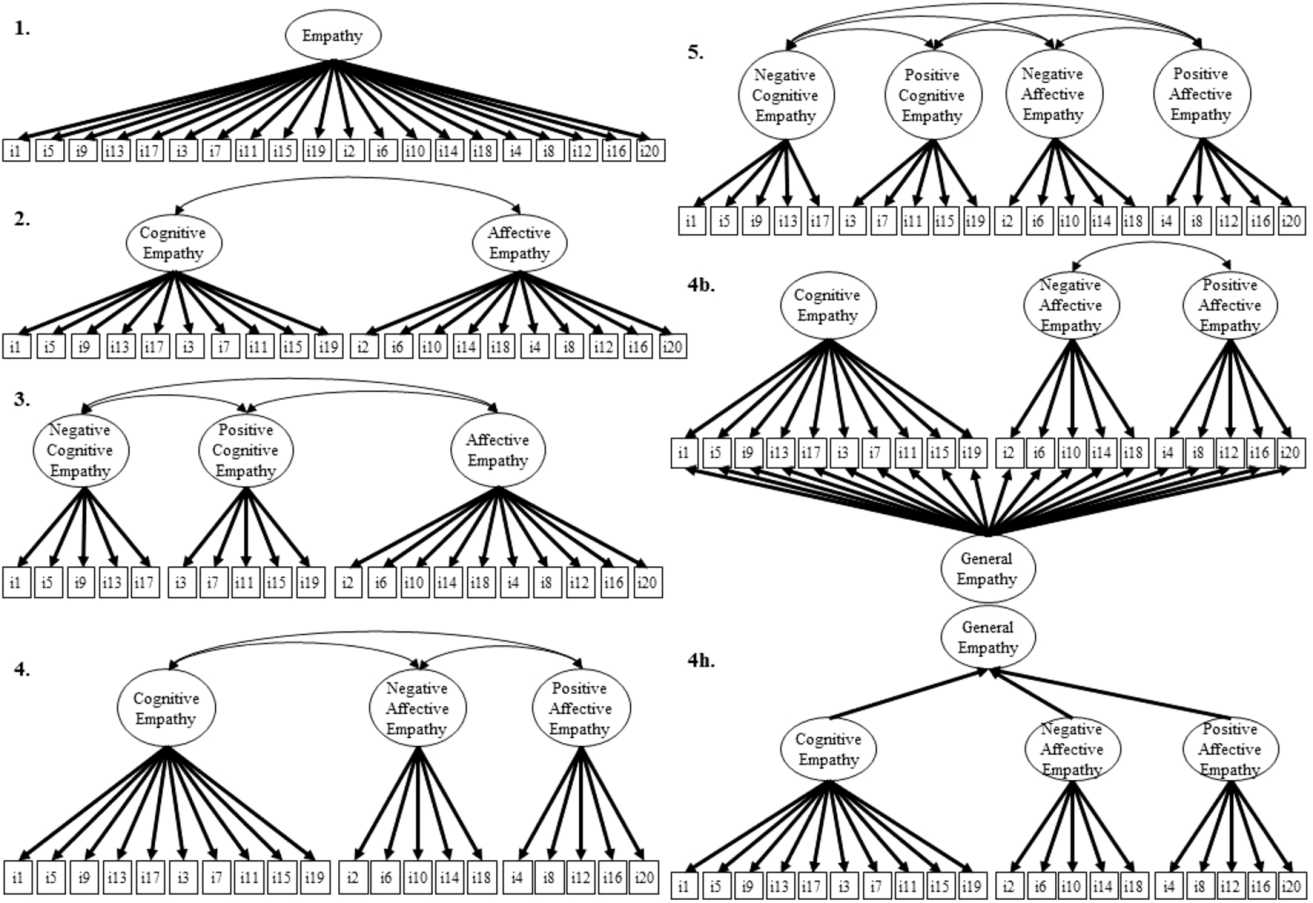
Models tested in the current study. Models 1–5. Squares indicate item numbers, and ellipses indicate latent factors. Item error terms are not displayed. b, bifactor model; h, hierarchical model.

Confirmatory Factor Analyses (CFA) were performed using the lavaan package (48) for R version 4.0.1. CFA was conducted using Weighted Least Squares Mean and Variances (WLSMV) estimation, as the 20 items were treated as ordinal variables. Although Likert scales are commonly treated as interval scales, they are technically ordinal scales (49); and it is recommended to use WLSMV for Likert scales regardless of the number of categories (50). We tested all the seven theoretical models suggested in the original study Brett, Becerra (13). These models were as follows: Model 1 was a unidimensional model, in which all 20 items were associated with a single “general empathy” factor. Model 2 was a two-factor model differentiating cognitive and affective empathy. Model 3 was a three-factor model dividing cognitive empathy into positive and negative valences. Model 4 was an alternative three-factor model that distinguished the positive and negative valences of affective empathy. Model 5 was based on positive and negative valence, which includes four factors (positive cognitive empathy, negative cognitive empathy, positive affective empathy and negative affective empathy). Finally, model (4h) that presented a hierarchical version of model 4 and model (4b) that was a bifactor three-factor model version of the same model were tested as well (see **Figure 1**).

The fit of models was evaluated using three key indices: Comparative Fit Index (CFI), the Root Mean Square Error of Approximation (RMSEA), and the Standardized Root Residual (SRMR). For these indices, acceptable fit values are defined as follows: CFI values should be greater than 0.90 (51, 52), RMSEA values should be less than 0.08 and SRMR should be less than 0.1 (53). Factor loadings greater than 0.32 were considered indicative of meaningful loadings (54).

To examine the extent of multi-dimensionality in the PES, model-based indices of the bifactor model were calculated using Dueber (55) calculator. The explained common variance of the general factor (ECV) is the proportion of the common variance the general factor explains (i.e., overall empathy). Omega Hierarchical (*ω_H_*) was calculated to provide the percentage of systematic variance attributed to a general empathy factor, after accounting for the variance attributed by the specific factors (e.g., cognitive empathy). A measure may be interpreted as essentially unidimensional when ECV values ≥.60 and *ω_H_* values ≥.70, or *ω_H_* values ≥.80 (56–58).

Cronbach’s alpha (α) and McDonald’s Omega (ω) were calculated to assess internal consistency. The values greater than 0.90 indicate excellent consistency, while those greater than 0.80 and 0.70 are considered good and acceptable, respectively (59).

Pearson correlation coefficients were calculated to assess concurrent and discriminant validity. We expected the PES scores to show a positive correlation with QCAE and both subscales of IRI (perspective taking and empathic concern) indicating concurrent validity. It was anticipated that the cognitive empathy factor from the PES would correlate more strongly with the cognitive empathy factors from the QCAE and IRI compared with their affective empathy factors, and vice versa for the affective empathy PES factors.

Since individuals with alexithymia struggle to identify and describe emotions (60), we anticipated that high levels of alexithymia correlate with lower levels of empathy. According to the self-other model of empathy, the impairments in the affective representation system cause alexithymia which negatively impacts empathy processing (14). In a recent meta-analysis of the association between social anxiety and empathy, Pittelkow, Aan Het Rot (8) highlighted the complexity and inconsistency in this relationship. Their analysis revealed an overall positive association between social anxiety and affective empathy, while the link between social anxiety and cognitive empathy was less clear, showing a small negative correlation only in clinical samples. Therefore, we expected to find a positive correlation between the SPIN and PES affective empathy.

We conducted multigroup CFA to test the measurement invariance of the PES between the Iranian and Australian sample. The measurement invariance testing was performed in four steps. First, single-group CFAs were investigated for both groups separately to establish baseline models. Next, configural invariance with multigroup CFAs was examined to ensure that the factor structure was consistent across both samples. Then, the metric invariance was performed with restricting all factor loadings to be equal across both samples. The last step was testing scalar measurement invariance by restricting all intercepts to be equal across both groups. Model comparisons were made based on CFI differences. A change in ΔCFI of 0.01 or more indicates a significant decline in model fit, suggesting non-invariance (61).

## Results

### Descriptive statistics and reliability coefficients

Descriptive statistics and reliability coefficients for all PES subscales and the total scale score are displayed in **Table 1**. All the PES subscales and the total score showed good and acceptable alpha and omega coefficients (α range from 0.73 to 0.88, and ω range from 0.74 to 0.88), except negative affective empathy scale (α and ω = 0.67).

**Table 1:** Descriptive statistics and Cronbach’s alpha and McDonald’s omega reliability coefficients for the administered measures.

Measure/Subscale	M	SD	α	ω
PES				
Cognitive empathy	34.82	7.38	.88	.88
Affective empathy	26.73	6.16	.76	.76
Negative affective empathy	11.96	3.47	.67	.67
Positive affective empathy	14.76	3.87	.73	.74
Total PES	61.55	11.60	.80	.87
QCAE				
Cognitive empathy	56.03	7.14	.80	.80
Affective empathy	32.30	4.10	.44	.54
IRI				
Perspective taking	13.61	3.72	.71	.73
Empathic concern	19.47	3.06	.61	.63
PAQ				
N-DIF	14.13	5.35	.74	.76
PDIF	11.10	4.81	.79	.80
N-DDF	16.40	6.44	.85	.86
P-DDF	12.90	5.83	.85	.85
G-EOT	23.98	9.18	.84	.85
SPIN				
Fear	4.54	4.16	.81	.83
Avoidance	5.01	4.86	.84	.85
physiological	3.33	2.97	.74	.74

No skewness or kurtosis values exceeded ±2. Note. α, Cronbach’s alpha; ω, McDonald’s Omega; PES, Perth Empathy Scale; QCAE, Questionnaire for Cognitive and Affective Empathy; IRI, Interpersonal Reactivity Index; PAQ, Perth Alexithymia Questionnaire; N-DIF, difficulty identifying negative feelings; PDIF, difficulty identifying positive feelings; N-DDF, difficulty describing negative feelings; P-DDF, difficulty describing positive feelings; G-EOT, general externally oriented thinking; SPIN, Social Phobia Inventory.

### Factor structure

Fit indices for all CFA models are presented in **Table 2**. The unidimensional model exhibited the poorest fit compared to the other models, indicating that the PES assesses a multidimensional construct. The fit indices suggested that distinguishing affective empathy into negative and positive valences provided a better fit than distinguishing cognitive empathy by valences. In Model 3 and Model 5, the positive and negative empathy factors were highly correlated (*r* = .96), closely mirroring the finding from the original study, where a correlation of.97 between negative and positive empathy was observed, suggesting that these factors capture the same latent construct. Consequently, Model 4, which does not differentiate positive and cognitive empathy, emerged as the most appropriate model. Moreover, the bifactorial model showed a superior fit compared to the hierarchical model (4h). However, the model-based indices of model 4b suggested that the scale should not be interpreted as an essentially unidimensional measure with greater focus on the subscales, *ω_H_* = .61 & ECV = .43. Additionally, the standardized item factor loadings are presented in **Table 3**.

**Table 2:** Goodness-of-fit index values from confirmatory factor analyses of the PES.

Models	χ^2^ (df)	RMSEA	CFI	TLI	SRMR
1	1232.60 (170)	.085	.910	.899	.091
2	11984.865 (190)	.057	.960	.955	.066
3	619.927 (167)	.056	.962	.956	.065
4	496.578 (167)	.048	.972	.968	.057
5	422.667 (164)	.043	.978	.975	.053
4b	382.48 (150)	.042	.980	.975	.050
4h	429.006 (149)	.047	.972	.967	.056

PES, Perth Empathy Scale; df, degrees of freedom; RMSEA, root mean square error approximation; CFI, comparative fit index; SRMR, standardized root mean square residual.

**Table 3:** Standardized item factor loadings for all PES items and subscales.

	Model 4	Bifactor Model 4	
	*ʎ*	*ʎ_Gen_*	*ʎ_Sp_*
CE			
1. Just by seeing or hearing someone, I know if they are feeling sad.	.54	.27	.53
5. Just by seeing or hearing someone, I know if they are feeling angry.	.58	.29	.57
9. Just by seeing or hearing someone, I know if they are feeling scared.	.70	.40	.60
13. Just by seeing or hearing someone, I know if they are feeling disgusted.	.71	.49	.49
17. Just by seeing or hearing someone, I know if they are feeling embarrassed.	.66	.38	.56
3. Just by seeing or hearing someone, I know if they are feeling happy.	.59	.35	.49
7. Just by seeing or hearing someone, I know if they are feeling amused.	.65	.47	.43
11. Just by seeing or hearing someone, I know if they are feeling calm.	.71	.53	.44
15. Just by seeing or hearing someone, I know if they are feeling enthusiastic.	.76	.58	.47
19. Just by seeing or hearing someone, I know if they are feeling proud.	.66	.40	.54
NAE			
2. When I see or hear someone who is sad, it makes me feel sad too	.50	.35	.24
6. When I see or hear someone who is angry, it makes me feel angry too.	.54	.32	.48
10. When I see or hear someone who is scared, it makes me feel scared too.	.53	.33	.41
14. When I see or hear someone who is disgusted, it makes me feel disgusted too.	.61	.39	.48
18. When I see or hear someone who is embarrassed, it makes me feel embarrassed too.	.45	.26	.48
PAE			
4. When I see or hear someone who is happy, it makes me feel happy too.	.51	.40	.58
8. When I see or hear someone who is amused, it makes me feel amused too.	.56	.58	.97
12. When I see or hear someone who is calm, it makes me feel calm too.	.66	.59	.27
16. When I see or hear someone who is enthusiastic, it makes me feel enthusiastic too.	.68	.60	.37
20. When I see or hear someone who is proud, it makes me feel proud too.	.57	.52	.15

standardized factor loadings for factors; CE, negative empathy; NAE, negative affective empathy; PAE, positive affective empathy.

### Measurement invariance

The measurement invariance of the best model (model 4) was evaluated across cultures. The configural model showed acceptable fit indices. Equality constraints were subsequently applied to all factor loadings and the ΔCFI indicated full metric invariance. Next, equality constraints were imposed on all item intercepts to assess scalar invariance. However, at the scalar level, the ΔCFI exceeded the.01 criterion, indicating non-invariance. Inspection of the modification indices suggested that freeing the constraints for items 1, 8, and 18 would improve the fit of the model. As can be seen in **Table 4**, after doing so, the ΔCFI (= .009) indicated partial scalar invariance for culture. The intercepts for items 1 (i.e., cognitive empathy for sadness) and 18 (i.e., affective empathy for embarrassment) were higher for Iranian participants (b = 3.86 and 1.79, respectively) compared to Australian participants (b = 3.51 and 2.35, respectively), while the intercept for item 8 (i.e., affective empathy for amusement) was higher for Australian participants (b = 3.16) than for Iranians (b = 2.36).

**Table 4:** Measurement invariance for the model 4 across culture.

Models	χ^2^ (df)	CFI	RMSEA	SRMR	ΔCFI
Configural	888.75 (334)	.977	.054	.054	---
Metric	1079.80 (351)	.970	.056	.059	-.007
Scalar	1632.84 (368)	.948	.069	.071	-.022
Partial Scalar	1318.64 (365)	.961	.057	.065	-.009

#### Relationships with other constructs/measures

Pearson correlations between the PES and other measures are shown in **Table 5**. As expected higher levels of empathy were correlated with higher levels of the QCAE and the IRI. Affective and cognitive empathy of PES were correlated with affective and cognitive empathy of IRI and QCAE, respectively. Most of PAQ subscales showed significant negative correlations with PES. The subscales of the SPIN showed significant correlation with PES subscales. Specifically, the avoidance subscale of SPIN demonstrated a negative correlation with both negative and affective empathy.

**Table 5:** Pearson correlations for scales of the PES with the QCAE, the IRI, SPIN, PAQ.

		CE	AE	NAE	PAE
QCAE					
	Cognitive empathy	.54^**^	.25^**^	.10^**^	.30^**^
	Affective empathy	.05	.38^**^	.45^**^	.21^**^
IRI					
	Perspective taking	.33^**^	.17^**^	.05	.23^**^
	Empathic concern	.17^*^	.24^**^	.23^**^	.17^**^
PAQ					
	N-DIF	-.13^**^	.14^**^	.25^**^	-.00
	P-DIF	-.17^**^	.04	.18^**^	-.10^**^
	N-DDF	-.13^**^	.11^**^	.21^**^	-.01
	P-DDF	-.15^**^	.04	.17^**^	-.09^*^
	G-EOT	-.14^**^	-.00	.09^*^	.08^**^
SPIN					
	Fear	-.07^*^	.13^**^	.31^**^	-.06
	Avoidance	.07^*^	-.08^**^	-.24^**^	-.08^**^
	Physiological	-.01	.17^**^	.31^**^	-.00

PES, Perth Empathy Scale; CE, Cognitive empathy; AE, Affective empathy; NAE, Negative affective empathy; PAE, positive affective empathy; QCAE, Questionnaire for Cognitive and Affective Empathy; IRI, Interpersonal Reactivity Index; N-DIF, Negative-Difficulty identifying feelings, P-DIF, Positive-Difficulty identifying feelings; N-DDF, Negative-Difficulty describing feelings; P-DDF, Positive-Difficulty describing feelings; G-EOT, General-Externally orientated thinking. *Indicates p <.05. **indicates p <.001.

## Discussion

The current study aimed to examine the psychometric properties of the Persian version of the Perth Empathy Scale (PES) in an Iranian sample. The results provided strong evidence supporting the validity and reliability of the PES for measuring empathy across affective and cognitive dimensions, also distinguishing between positive and negative emotional valences. These findings contribute to the research on empathy measurement by demonstrating the applicability of the PES in non-Western cultures, expanding its cross-cultural utility.

To the best of our knowledge, three studies in China, Australia and Poland have assessed the psychometric features of the PES (13, 29, 30, 32). The scale is relatively new and, only a limited number of studies have examined its psychometric characteristics, although all studies have provided evidence that the PES exhibits good reliability and validity to assess empathy across multiple cultures. In terms of the factorial structure, the three-factor model was identified as the most appropriate model, replicating findings from previous studies (13, 30, 32). However, as with previous studies (13, 29–32), the present results suggest that there may be some utility for the four-factor model. As with previous studies (13, 30, 32), a bifactor model provided the best, numerical, model fit, although model-based indices (i.e., ECV & ωH) suggested that the subscales should be used to interpret PES scores. This is in line with the theoretical underpinnings of the PES aligning with the theoretical framework suggesting that empathy includes both cognitive and emotional components and highlight the importance of viewing empathy as a multidimensional construct.

Crucially, distinguishing between emotional valences is highlighted in recent theories and models as an important factor in empathy, as it allows a more nuanced understanding of the empathic experience (3, 13). Despite the theoretical importance prior empathy measures –such as the Interpersonal Reactivity Index (IRI) and the Questionnaire of Cognitive and Affective Empathy (QCAE), have often lacked the capacity to adequately separate cognitive from affective empathy or to distinguish between emotions of varying valences.

The internal consistency of the PES was generally high, as indicated by Cronbach’s alpha and McDonald’s omega coefficients. The only exception was the Negative Affective Empathy (NAE) subscale, which exhibited somewhat lower reliability, suggesting that this subscale may require further refinement in future research. However, it should be noted that the NAE subscale provided higher reliability than the affective empathy scale of the QCAE and the empathy concern scale from the IRI. Concurrent validity of the PES was confirmed through expected significant correlations with established empathy measures, such as the QCAE and IRI. As expected, the cognitive empathy subscales of the PES showed stronger correlations with the cognitive empathy subscales of the QCAE and IRI, while the affective empathy subscales demonstrated stronger correlations with measures of affective empathy. The PES also displayed significant negative correlations with alexithymia, particularly among individuals with difficulties in identifying and describing emotions, further supporting the scale’s discriminant validity. These findings underscore the PES’s ability to capture the complexity of empathy as it relates to one’s own emotional awareness (i.e., alexithymia).

Regarding the relationship between empathy and social anxiety, individuals with higher levels of social avoidance reported lower scores of positive and negative affective empathy. Contrastingly, individuals with higher levels of fear of social settings, and physiological characteristics of social phobias reported greater negative affective empathy. Additionally, there were small correlation between the cognitive empathy and subscales of social anxiety. The results align with recent systematic reviews, which report that social anxiety is more closely associated with affective empathy than with cognitive empathy (8). The present results indicate that socially anxious individuals might be more attuned to the negative emotions of others, exacerbating their discomfort in social situations. This supports previous studies suggesting that symptoms like anxiety and depression are often driven by a predisposition to negative affect, which makes individuals more susceptible to psychopathology (29, 62).

Measurement invariance analysis was also conducted for the three-factor bifactor model to examine if the PES measures empathy similarly across Iranian and Australian adults, and full configural and metric invariance and partial scalar invariance were achieved. These results demonstrates that PES measures empathy similarly across these cultural groups, supporting the cross-cultural applicability of the scale. The PES items showing non-invariance asked about cognitive empathy for sadness, and affective empathy for embarrassment and amusement, suggesting that, after controlling for their underlying empathic tendencies, Iranians report a greater tendency to recognize sadness and feel another’s embarrassment, but have less tendency to feel another’s amusement, compared to westerners.

In addressing the lack of measurement invariance at the scalar level between Iranian and Australian groups, previous research provides relevant insights. Ranjbar, Mazidi (63), in their investigation using the Emotional Beliefs Questionnaire, found that Iranian participants scored significantly higher on items associated with beliefs about negative emotions compared to American participants. Similarly, another study (64) demonstrated that Iranians scored higher in the Negative-Activating Behavior domain of the Perth Emotion Regulation Competency Inventory. The findings of the present study are consistent with these prior observations, as Iranians scored higher on items related to sadness and embarrassment (1 and 18). Collectively, these results suggest that Iranians may experience negative emotions with greater intensity, highlighting potential cultural differences in emotional experience and expression.

To the best of our knowledge, this is the first study to examine the PES among an Iranian sample and to assess MI between Western and Asian samples. However, the study also has some limitations. The study did not include a clinical sample, which limits the ability to generalize these findings to individuals with clinically emotional difficulties. Future research could address this by examining the psychometric properties of the PES in clinical populations, providing insights into its utility for assessing empathy in individuals with psychological disorders, potentially enhancing the applicability of the PES in clinical settings. Due to the lack of access to participants for retest, test-retest was not assessed in this study, which constitutes another limitation of our research. Future studies are encouraged to examine the temporal consistency of the scale using the test-retest method.

In summary, the Persian version of the PES demonstrates strong psychometric properties, making it a valuable tool for assessing empathy in Iranian populations. The scale’s ability to differentiate between cognitive and affective dimensions of empathy, as well as between positive and negative emotions, provides a comprehensive assessment of empathy that can inform both research and clinical practice. While showing greater reliability than other scales, refinement of the Negative Affective Empathy (NAE) subscale may provide greater future applications. Future studies should further explore the applicability of the PES in other cultural contexts and assess its utility in clinical interventions aimed at improving empathy in various psychological disorders.

## Data Availability

The original contributions presented in the study are included in the article/**Supplementary Material**. Further inquiries can be directed to the corresponding author.
