# Supplementary material for: Measuring cognitive and affective empathy across positive and negative emotions: psychometric properties and measurement invariance of the Perth Empathy Scale
Source: Front Psychiatry. 2025 Mar 11;16:1533611. doi: 10.3389/fpsyt.2025.1533611 (PMC11932917; doi:10.3389/fpsyt.2025.1533611)
Supplement: Supplementary file 1 [file Table1.pdf]

Supplementary Tables.

Table 1. Measurement invariance for the bifactor model across culture

Models	χ^2 (df)	CFI	RMSEA	SRMR	ΔCFI
Configural	677.27(300)	.984	.039	.046	---
Metric	949.09(336)	.974	.047	.055	-.009
Scalar	1094.90(352)	.969	.051	.059	-.005
Strict	1298.84(372)	.961	.055	.066	-.006
