# Supplementary material for: Measuring cognitive and affective empathy across positive and negative emotions: psychometric properties and measurement invariance of the Perth Empathy Scale
Source: Front Psychiatry. 2025 Mar 11;16:1533611. doi: 10.3389/fpsyt.2025.1533611 (PMC11932917; doi:10.3389/fpsyt.2025.1533611)
Supplement: Supplementary file 2 [file Table2.pdf]

The Persian version of Perth Empathy Scale

پرسشنامه همدلی پرس

این پرسشنامه در مورد این است که شما چقدر راحت احساسات دیگران را متوجه می‌شوید و تجربه می کنید است. لطفاً به هرکدام از آیتم‌ها با استفاده از مقیاس موجود پاسخ دهید.

گزاره	تقریباً هیچ وقت	بعضی اوقات	حدود نیمی از اوقات	بیشتر اوقات	تقریباً همیشه
۱. دیدن کسی یا شنیدن صدایش برایم کافی است که متوجه شوم احساس غمگینی می کند یا نه.					
۲. وقتی کسی که غمگین است را می‌بینم یا صدایش را می‌شنوم، باعث می‌شود من هم احساس غمگینی کنم.					
۳. دیدن کسی یا شنیدن صدایش برایم کافی است که متوجه شوم احساس خوشحالی می‌کند یا نه.					
۴. وقتی کسی که خوشحال است را می‌بینم یا صدایش را می‌شنوم، باعث می‌شود من هم احساس خوشحالی کنم.					
۵. دیدن کسی یا شنیدن صدایش برایم کافی است که متوجه شوم خشمگین هست یا نه.					
۶. وقتی کسی که خشمگین است را می‌بینم یا صدایش را می‌شنوم، باعث می شود من هم احساس خشم کنم.					
۷. دیدن کسی یا شنیدن صدایش برایم کافی است که متوجه شوم احساس تفریح و سرگرمی می‌کند یا نه					

۸. وقتی کسی که سرگرم است را می‌بینم یا صدایش را می‌شنوم، باعث می شود من هم احساس سرگرمی کنم.					
۹. دیدن کسی یا شنیدن صدایش برایم کافی است که متوجه شوم احساس ترس می‌کند یا نه					
۱۰. وقتی کسی که ترسیده است را می‌بینم یا صدایش را می‌شنوم، باعث می‌شود من هم احساس ترس کنم.					
۱۱. دیدن کسی یا شنیدن صدایش برایم کافی است که متوجه شوم احساس آرامش می‌کند یا نه					
۱۲. وقتی کسی که آرام است را می‌بینم یا صدایش را می‌شنوم، باعث می‌شود من هم احساس آرامش کنم.					
۱۳. دیدن کسی یا شنیدن صدایش برایم کافی است که متوجه شوم احساس نفرت می‌کند یا نه					
۱۴. وقتی کسی که متنفر است را می‌بینم یا صدایش را می‌شنوم، باعث می‌شود من هم احساس تنفر کنم.					
۱۵. دیدن کسی یا شنیدن صدایش برایم کافی است که متوجه شوم احساس اشتیاق می‌کند یا نه.					
۱۶. وقتی کسی که مشتاق است را می‌بینم یا صدایش را می‌شنوم، باعث می‌شود من هم احساس اشتیاق کنم.					
۱۷. دیدن کسی یا شنیدن صدایش برایم کافی است که متوجه شوم احساس خجالت می‌کند یا نه					
۱۸. وقتی کسی که خجالت می‌کشد را می‌بینم یا صدایش را می‌شنوم، باعث می‌شود من هم احساس خجالت کنم.					
۱۹. دیدن کسی یا شنیدن صدایش برایم کافی است که متوجه شوم احساس غرور و افتخار می‌کند یا نه.					

۲۰. وقتی کسی که احساس غرور دارد را می‌بینم یا صدایش را می‌شنوم، باعث می‌شود من هم احساس غرور و افتخار کنم.					

جدول نمره گذاری پرسشنامه همدلی پرس

خرده مقیاس ها	
همدلی شناختی منفی	۱۷-۱۳-۹-۵-۱
همدلی شناختی مثبت	۱۹-۱۵-۱۱-۷-۳
همدلی عاطفی منفی	۱۸-۱۴-۱۰-۶-۲
همدلی عاطفی مثبت	۲۰-۱۶-۱۲-۸-۴
همدلی عاطفی کلی	جمع همدلی عاطفی مثبت و منفی
همدلی شناختی کلی	جمع همدلی شناختی مثبت و منفی
نمره کل همدلی	جمع هم آیتم ها
