# Supplementary material for: Measuring cognitive and affective empathy across positive and negative emotions: psychometric properties and measurement invariance of the Perth Empathy Scale
Source: Front Psychiatry. 2025 Mar 11;16:1533611. doi: 10.3389/fpsyt.2025.1533611 (PMC11932917; doi:10.3389/fpsyt.2025.1533611)
Supplement: Supplementary file 3 [file Table3.pdf]

Table 3. Perth Empathy Scale

This questionnaire asks about how easily you recognise and experience the emotions of others. Please score the following statements using the scale provided. Circle one answer for each statement.

		**Almost never**	**Sometimes**	**About half the time**	**Most of the time**	**Almost always**
1	Just by seeing or hearing someone, I know if they are feeling sad.	1	2	3	4	5
2	When I see or hear someone who is sad, it makes me feel sad too.	1	2	3	4	5
3	Just by seeing or hearing someone, I know if they are feeling happy.	1	2	3	4	5
4	When I see or hear someone who is happy, it makes me feel happy too.	1	2	3	4	5
5	Just by seeing or hearing someone, I know if they are feeling angry.	1	2	3	4	5
6	When I see or hear someone who is angry, it makes me feel angry too.	1	2	3	4	5
7	Just by seeing or hearing someone, I know if they are feeling amused.	1	2	3	4	5
8	When I see or hear someone who is amused, it makes me feel amused too.	1	2	3	4	5
9	Just by seeing or hearing someone, I know if they are feeling scared.	1	2	3	4	5
10	When I see or hear someone who is scared, it makes me feel scared too.	1	2	3	4	5

11	Just by seeing or hearing someone, I know if they are feeling calm.	1	2	3	4	5
12	When I see or hear someone who is calm, it makes me feel calm too.	1	2	3	4	5
13	Just by seeing or hearing someone, I know if they are feeling disgusted.	1	2	3	4	5
14	When I see or hear someone who is disgusted, it makes me feel disgusted too.	1	2	3	4	5
15	Just by seeing or hearing someone, I know if they are feeling enthusiastic.	1	2	3	4	5
16	When I see or hear someone who is enthusiastic, it makes me feel enthusiastic too.	1	2	3	4	5
17	Just by seeing or hearing someone, I know if they are feeling embarrassed.	1	2	3	4	5
18	When I see or hear someone who is embarrassed, it makes me feel embarrassed too.	1	2	3	4	5
19	Just by seeing or hearing someone, I know if they are feeling proud.	1	2	3	4	5
20	When I see or hear someone who is proud, it makes me feel proud too.	1	2	3	4	5
